# ﻿Non-lichenized *Cytosporella*, including *C.fuligomixta* sp. nov., and related plant-associated and fungicolous genera are close to foliicolous, lichenized fungi (Ascomycota, Graphidales)

**DOI:** 10.3897/mycokeys.115.138252

**Published:** 2025-03-06

**Authors:** Marcin Piątek, Monika Stryjak-Bogacka, Paweł Czachura

**Affiliations:** 1 W. Szafer Institute of Botany, Polish Academy of Sciences, Lubicz 46, PL-31-512 Kraków, Poland W. Szafer Institute of Botany, Polish Academy of Sciences Kraków Poland

**Keywords:** Ascomycota, Graphidales, Gomphillaceae, one new species, one new subfamily, sooty mould communities, taxonomy

## Abstract

The genus *Cytosporella* includes non-lichenized, plant associated fungi producing eustromatic conidiomata, phialidic conidiophores and hyaline, ellipsoid conidia. Of the 69 names assigned to this genus in Index Fungorum, only three species are associated with sequence data. In this study, a new species: *Cytosporellafuligomixta* is described based on a strain isolated from the sooty mould community on *Quercusrobur* leaves in Poland. The phylogenetic analyses including sequences of two loci (LSU, mtSSU) showed that *Cytosporella* species, together with members of four other non-lichenized, plant associated or fungicolous genera, namely *Cladosterigma*, *Neoacrodontiella*, *Nothoramularia* and *Vanderaaea*, form a sister group to lichenized and lichenicolous fungi from the family Gomphillaceae and order Graphidales. Previously, *Cladosterigma* was resolved as a member of Gomphillaceae using multi-locus (mtSSU, SSU, LSU, ITS, *rpb2*, *tef1*) and two-locus (LSU, mtSSU) sequence analyses, while *Cytosporella*, *Neoacrodontiella*, *Nothoramularia* were shown to belong to this family using LSU sequence analyses. However, none of them resolved these genera as a sister group to lichenized members of Gomphillaceae. The placement of the genus *Vanderaaea* within Gomphillaceae is shown here for the first time. Due to phylogenetic, morphological and ecological characteristics a new subfamily Cladosterigmoideae is described for these five non-lichenized genera.

## ﻿Introduction

The genus *Cytosporella* includes non-lichenized, plant associated fungi producing eustromatic conidiomata, phialidic conidiophores and hyaline, ellipsoid conidia (Sutton 1980; Crous et al. 2019b; Li et al. 2020). Index Fungorum (2024) includes 69 names assigned to *Cytosporella* but DNA sequence data are available only for three species: *Cytosporellacalamagrostidis*, *C.chamaeropis* and *C.juncicola*. The type species is *Cytosporellasycina* (Clements and Shear 1931; Sutton 1980; Li et al. 2020). It has been described from branches of *Ficuscarica* in France (Saccardo 1880) but not sequenced yet, making current taxonomy of the genus tentative. The species of *Cytosporella* are saprobic or parasitic on different hosts, mostly on branches of deciduous trees (Li et al. 2020; species data in Index Fungorum 2024). During our surveys of sooty mould communities on ornamental woody plants in southern Poland a new species of *Cytosporella* (described here as *Cytosporellafuligomixta*) was isolated from a sooty mould colony on leaves of *Quercusrobur* (Fagaceae).

The sequenced species of *Cytosporella* were shown to be related to three non-lichenized, plant associated or fungicolous genera: *Neoacrodontiella*, *Nothoramularia* and *Vanderaaea*, and altogether they were assigned to the family Acarosporaceae and order Acarosporales (Crous et al. 2019a, 2019b, 2021, 2023). This order and family contain saxicolous and terricolous lichenized fungi in the subclass Acarosporomycetidae of the Lecanoromycetes (Reeb et al. 2004; Miadlikowska et al. 2014; Westberg et al. 2015). Our initial query of sequences of *Cytosporella* (including new species *C.fuligomixta*), *Neoacrodontiella*, *Nothoramularia* and *Vanderaaea* in GenBank showed that most closely related sequences belong to members of the lichenized family Gomphillaceae. Also, non-lichenized, fungicolous *Cladosterigmaclavariellum*, which was recently included in Gomphillaceae (Guterres et al. 2020), was amongst the resultant related sequences. This family includes mostly foliicolous lichens and is included either in Graphidales or Ostropales within subclass Ostropomycetidae of the Lecanoromycetes (Baloch et al. 2010; Miadlikowska et al. 2014; Kraichak et al. 2018). Indeed, a recent phylogenetic tree, based on LSU sequences, published in Crous et al. (2024), placed *Cladosterigma*, *Cytosporella*, *Neoacrodontiella* and *Nothoramularia* inside the family Gomphillaceae. The genus *Vanderaaea* was not included in these analyses. Similarly, multi-locus (using mtSSU, SSU, LSU, ITS, *rpb2* and *tef1* sequences) and two-locus (using LSU and mtSSU sequences) trees, published by Guterres et al. (2020), placed *Cladosterigma* inside the family Gomphillaceae with *Vezdamycesvulgaris* (syn. *Gyalideopsisvulgaris*) being the most basal species. However, in these cases (based on GenBank accession numbers of sequences included by Guterres et al. 2020 in their tables), sequences of “*Gyalideopsisvulgaris*” used in the multi-locus tree belonged to *Gyalideapraetermissa* that is a member of Gyalectaceae (Ertz et al. 2021), while LSU sequence used in two-locus tree belonged to other species (closest hits in GenBank are species of Eurotiomycetes) that could have been responsible for such a result.

In this study, we describe and illustrate a new species of *Cytosporella* isolated from a sooty mould colony on leaves of *Quercusrobur*. Additionally, based on publicly available sequences, we reassess phylogenetic placement of species of *Cladosterigma*, *Cytosporella*, *Neoacrodontiella*, *Nothoramularia* and *Vanderaaea* using two-locus (LSU, mtSSU) sequence analyses.

## ﻿Materials and methods

### ﻿Strain and morphological analyses

The strain was obtained from sooty mould communities during the study of sooty moulds on ornamental woody plants cultivated in municipal greenery in southern Poland (Piątek et al. 2023). Macroscopic features of cultures were documented using 2-week-old colonies grown on malt extract agar (MEA – Blakeslee’s formula), potato dextrose agar (PDA) and oatmeal agar (OA) at 6 °C, 15 °C and 25 °C. Growth at different temperatures was assessed by measuring the colony diameter after 2 weeks and 4 weeks. Microscopic features were studied using colonies grown on MEA and OA at 15 °C after 7 weeks and 6 weeks, respectively. The characteristics of hyphae were observed on MEA and characteristics of conidiomata, conidiophores and conidia were studied on OA. Hyphae taken from the edge of the colony and mature conidiomata were mounted in lactic acid (80%) on microscope slides and analysed under Nikon Eclipse 80i light microscope. Microscopic structures were measured and photographed using NIS‐Elements BR 3.0 imaging software. Holotype is a dried specimen obtained from culture and is stored in the fungal collection of the W. Szafer Institute of Botany, Polish Academy of Sciences, Kraków (KRAM F). Culture is deposited in the culture collection of the Westerdijk Fungal Biodiversity Institute (CBS) and in the W. Szafer Institute of Botany, Polish Academy of Sciences, Kraków.

### ﻿DNA isolation, amplification and sequencing

Genomic DNA was extracted from four-week-old MEA culture using DNeasy® Plant Mini Kit (Qiagen, Germany), according to the manufacturer’s protocol. A total of five loci were amplified: ITS1‐5.8S‐ITS2 rRNA (= ITS), partial large subunit rRNA (28S D1–D2 = LSU), small subunit mtRNA (=mtSSU), partial DNA-directed RNA polymerase II second largest subunit (*rpb2*) and translation elongation factor 1-alpha (*tef1*). To amplify the regions of ITS, LSU, mtSSU, *rpb2* and *tef1*, five different primer pairs were used, namely ITS1–ITS4 (White et al. 1990), LSU1Fd–LR5 (Vilgalys and Hester 1990; Crous et al. 2009), mrSSU1–mrSSU3R (Zoller et al. 1999), fRPB2-5F–fRPB2-7cR (Liu et al. 1999) as well as EF1-983F and EF1-2218R (Rehner and Buckley 2005), respectively. Polymerase chain reaction mixtures were performed in a total volume of 25 μL as explained in Piątek et al. (2023). Amplification conditions for ITS and LSU were described in Czachura et al. (2021), while conditions for subsequent loci were set as follows: an initial denaturation at 94 °C for 3 min, followed by 35 cycles (mtSSU, *rpb2*) or 40 cycles (*tef1*) of denaturation at 94 °C for 60 sec (mtSSU, *rpb2*) or 30 sec (*tef1*); annealing at 52 °C (mtSSU) or 54 °C (*rpb2*) or 55 °C (*tef1*) for 60 sec (mtSSU) or 90 sec (*rpb2*) or 50 sec (*tef1*); and extension at 72 °C for 1 min (mtSSU, *tef1*) or 2 min (*rpb2*). The process was finished with the final extension at 72 °C for 7 min (mtSSU) or 10 min (*rpb2*, *tef1*). PCR amplifications were confirmed on 1% agarose electrophoresis gels stained with SimplySafe (EURx, Poland). Amplicons were enzymatically cleaned using Exo-BAP Mix (EURx, Poland). The same primers as given above were used in sequencing reactions which were carried out by Macrogen Europe B.V. (Amsterdam, The Netherlands). Obtained sequences were assembled and trimmed in Geneious Prime 2020.0.4. Consensus sequences were deposited in the NCBI’s GenBank nucleotide database (https://www.ncbi.nlm.nih.gov/genbank/).

### ﻿Phylogenetic analyses

The affinities of obtained *Cytosporella* sequences and sequences of related genera and species were determined in the NCBIs GenBank nucleotide database using the megablast search tool (Zhang et al. 2000). For the phylogenetic analyses only LSU and mtSSU sequences were used due to limited sampling of reference sequences available for members of Gomphillaceae. The assembled concatenated LSU-mtSSU alignment contained available sequences of all sequenced species of *Cladosterigma*, *Cytosporella*, *Neoacrodontiella*, *Nothoramularia* and *Vanderaaea*, and sequences of selected members of Gomphillaceae and members of its most closely related family Graphidaceae used as an outgroup (Table 1). In the case of problematic sequences of *Vezdamycesvulgaris* (syn. *Gyalideopsisvulgaris*) used by Guterres et al. (2020), the correct sequences obtained from two different specimens were taken following Xavier-Leite et al. (2022).

**Table 1.** List of species, with country of origin, host/substrate, strain/voucher, GenBank accession numbers and references, used in phylogenetic analyses.

Species	Country	Host/substrate	Strain/voucher	GenBank acc. no.		References
				LSU	mtSSU	
*Aderkomycesheterellus*	Brazil	–	Cáceres & Aptroot 11953	KF833330	KF833342	I. Schmitt, T. Lumbsch, E. Kraichak (unpubl.)
*Asterothyriumlongisporum*	Costa Rica	–	Lücking s.n., F sample no. 4	AY341349	AY341363	Lücking et al. 2004
*Aulaxinaquadrangula*	Costa Rica	–	Lücking s.n., F sample no. 66	AY341350	AY341364	Lücking et al. 2004
*Aulaxinellaminuta*	Costa Rica	–	E. Baloch HK2 (GZU)	–	AY648887	Grube et al. 2004
*Chroodiscusdefectus*	Thailand	–	Papong 5118	FJ708490	FJ708497	–
*Cladosterigmaclavariellum*	Brazil	*Phyllachora* sp. on leaves of *Eugeniaflorida*	UB 23227	MK933757	MK910849	Guterres et al. 2020
*Cladosterigmaclavariellum*	Brazil	*Phyllachora* sp. on leaves of *Eugeniaflorida*	UB 23228	MK933758	MK910850	Guterres et al. 2020
*Clandestinotremastylothecium*	Nicaragua	–	Lücking 28636	JX421470	HQ639597	Rivas Plata et al. 2011, 2013
*Corticifragapeltigerae*	Luxembourg	Peltigeracf.rufescens	Marson 2015-05-02-1	KY462801	–	H.-O. Baral, G. Marson (unpubl.)
*Corticifragapeltigerae*	India	*Peltigeraelisabethae*	Zhurbenko 1353 (LE 260537)	KY661661	KY661684	Pino-Bodas et al. 2017
*Cruentotremathailandicum*	Thailand	–	Lumbsch 19955d	JF828975	JF828960	Rivas Plata and Lumbsch 2011
*Cytosporellacalamagrostidis*	Netherlands	*Calamagrostisarenaria*	CPC 46236	PP791461	–	Crous et al. 2024
*Cytosporellafuligomixta*	Poland	sooty mould community on *Quercusrobur* leaves	G107 = CBS 152343	PQ001665	PP999744	this study
*Cytosporellajuncicola*	USA	*Juncuseffusus*	CPC 38040	MN567660	–	Crous et al. 2019b
*Cytosporellachamaeropis*	Italy	*Chamaeropshumilis*	CBS 355.71	MH871929	–	Vu et al. 2019
*Dyplolabiaafzelii*	USA	–	Lücking 26509a	JX421483	JX421027	Rivas Plata et al. 2013
*Echinoplaca* sp.	Nicaragua	–	Lücking 28550	KF833328	KF833340	I. Schmitt, T. Lumbsch, E. Kraichak (unpubl.)
*Fissurinacomparimuralis*	El Salvador	–	Lücking 28103	JX421492	JX421042	Rivas Plata et al. 2013
*Fissurinamarginata*	Thailand	–	Lücking 24122	JX421493	HQ639613	Rivas Plata et al. 2011, 2013
*Fissurinarufula*	Fiji	–	Lumbsch 20521l	JX421497	JX421053	Rivas Plata et al. 2013
*Gomphilluscalycioides*	UK (Scotland)	–	Lumbsch 20100d (F)	KF833329	KF833341	I. Schmitt, T. Lumbsch, E. Kraichak (unpubl.)
*Gomphillusophiosporus*	Costa Rica	–	Will-Wolf 10006a (F)	AY341357	AY341371	Lücking et al. 2004
*Graphisscripta*	Germany	–	Staiger 982	DQ431937	AY648904	–
*Gyalectidiumcatenulatum*	Costa Rica	–	Lücking 032b	KF833323	KF833335	I. Schmitt, T. Lumbsch, E. Kraichak (unpubl.)
*Gyalectidiumimperfectum*	Costa Rica	–	Lücking s.n., F sample no. 2	AY341358	AY341372	Lücking et al. 2004
*Gyalideafritzei*	Sweden	–	Nordin 6022 (UPS)	HM244767	HM244744	Baloch et al. 2010
*Gyalideahyalinescens*	Costa Rica	–	AFTOL-ID 332	DQ973046	DQ972996	Miadlikowska et al. 2006
*Monocaleniamonospora*	Costa Rica	–	Lücking 032h	KF833327	KF833339	I. Schmitt, T. Lumbsch, E. Kraichak (unpubl.)
*Monocaleniamonospora*	Costa Rica	–	Lücking 032e	KF833325	KF833337	I. Schmitt, T. Lumbsch, E. Kraichak (unpubl.)
*Myriotremaolivaceum*	Australia	–	Lumbsch 19113f	EU075627	EU075579	–
*Neoacrodontiellaeucalypti*	Malaysia	*Eucalyptusurophylla*	CBS 145561	MK876437	–	Crous et al. 2019a
*Nothoramulariaragnhildianicola*	Germany	*Ragnhildianaferruginea* on leaves of *Artemisiavulgaris*	CBS 149076	OQ990069	–	Crous et al. 2023
*Ocellulariaoculata*	Australia	–	Mangold 33a	EU075613	EU075564	–
*Phaeographislobata*	Bermuda	–	Berger 19598	DQ431944	DQ431984	–
Psorotheciopsiscf.premneella	Cuba	–	Lücking et al. 41885b	MZ851727	–	Xavier-Leite et al. 2022
*Pycnotremapycnoporellum*	USA	–	Lücking 26545	HQ639658	HQ639584	Rivas Plata et al. 2011
*Rolueckiaaggregata*	Brazil	–	Cáceres & Aptroot 28665a	MZ851690	–	Xavier-Leite et al. 2022
*Rolueckiaconspersa*	Brazil	–	Xavier-Leite et al. 2803	MZ851644	–	Xavier-Leite et al. 2022
*Taitaiaaurea*	Kenya	Crocodiacf.clathrata	Kirika 5103 (EA)	MF372797	MF372799	Suija et al. 2018
*Taitaiaaurea*	Kenya	Crocodiacf.aurata	Rikkinen 16259	MF509277	–	Suija et al. 2018
*Thelotremasubtile*	Australia	–	Mangold 3j (F)	EU075651	EU075607	Mangold et al. 2008
*Thelotremasuecicum*	Turkey	–	Palice (ESS 21521)	AY300867	AY300917	Lumbsch et al. 2004
*Tricharialongispora*	Costa Rica	–	Lücking 033a	KF833326	KF833338	I. Schmitt, T. Lumbsch, E. Kraichak (unpubl.)
*Tricharialongispora*	Costa Rica	–	Lücking s.n., F sample no. 37	AY341360	AY341374	Lücking et al. 2004
*Vanderaaeaammophilae*	Netherlands	dead leaves of *Ammophilaarenaria*	CBS 886.68	MH878416	–	Crous et al. 2021
*Vezdamycesvulgaris*	Brazil/Costa Rica	–	Xavier-Leite 1476/AFTOL ID 105	MZ851481	AY584618	Lutzoni et al. 2004; Xavier-Leite et al. 2022

Sequence reads which we obtained were checked for quality and assembled. The complete LSU and mtSSU sequences were separately aligned for each single-gene dataset using MAFFT algorithm (Katoh et al. 2005) in Geneious 11.1.5. Phylogenetic trees were constructed by using the Maximum likelihood (ML) and Bayesian inference (BI) analysis. For both ML and BI analyses, the model of DNA evolution that best fitted the dataset was determined using the ModelTest-NG v. 0.2.0 under the Bayesian Information Criterion (BIC) (Darriba et al. 2020). Maximum likelihood (ML) analyses were performed using the RAxML-NG v. 1.1.1 (Kozlov et al. 2019). Branch support was inferred with 1000 bootstrap replicates. Bayesian analyses were conducted using a MrBayes v. 3.2.6 (Ronquist et al. 2012). One million generations were run, sampling every 100 generations. Four parallel chains, one cold and three heated, were used. A consensus tree was generated after discarding the first 25% of trees as burn-in. Average standard deviations of split frequencies dropped below 0.01 at the end of the runs. The final phylogenetic trees were graphically visualised using FigTree v.1.4.3.

## ﻿Results

### ﻿Phylogenetic analyses

**Figure 1.**
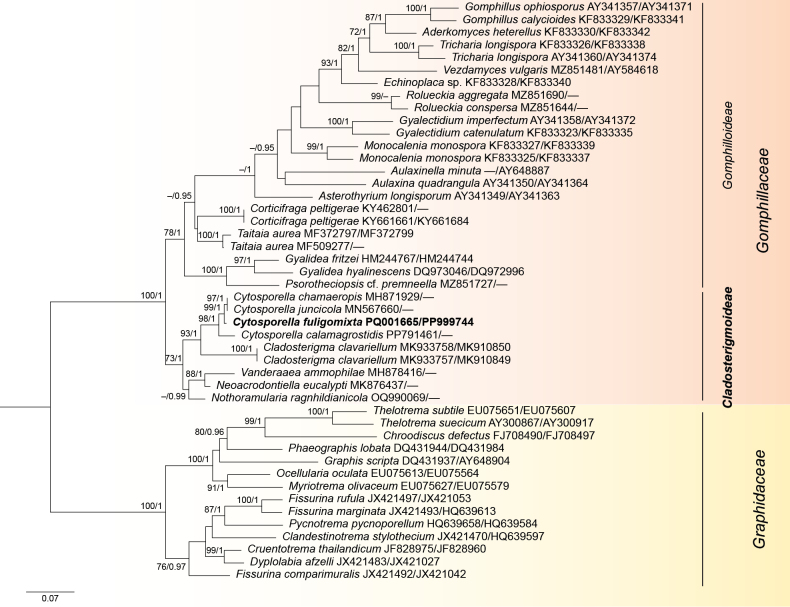
Phylogenetic tree of selected representatives of Gomphillaceae, including all sequenced species of *Cladosterigma*, *Cytosporella*, *Neoacrodontiella*, *Nothoramularia* and *Vanderaaea*, obtained from a maximum likelihood analysis of the combined two-locus alignment (LSU, mtSSU). Representatives of Graphidaceae are used as an outgroup. The positions of *Cytosporellafuligomixta* sp. nov. and Cladosterigmoideae subfam. nov. are indicated in bold. GenBank accession numbers (LSU/mtSSU) are given after species name. Numbers above branches indicate maximum likelihood bootstrap (MLB) support values > 70% and Bayesian posterior probabilities (BPP) > 0.95, respectively (MLB/BPP). The scale bar represents the expected number of changes per site.

The concatenated LSU-mtSSU alignment contained sequences belonging to 41 species. The alignment comprised a total of 1779 characters (LSU: 969, mtSSU: 810), including alignment gaps. The best matching substitution models selected for single locus alignments in the ML analysis were as follows: TIM2+I+G4 for LSU and TPM3uf+I+G4 for mtSSU. The BI analysis was performed with the following substitution model: GTR+I+G4 for LSU and mtSSU. ML and BI analyses resulted in similar tree topologies. The best scoring maximum likelihood phylogenetic tree is shown on Fig. 1. Maximum likelihood bootstrap (MLB) support values above 70% and Bayesian posterior probabilities (BPP) above 0.95 are shown at the nodes.

Representatives of the family Gomphillaceae formed strongly supported monophyletic lineage (MLB/BPP = 100/1). The strain of the new *Cytosporella* species clustered with members of the genus *Cytosporella* as sister to *C.chamaeropis* and *C.juncicola* (MLB/BPP = 98/1). Representatives of the genera *Cladosterigma*, *Cytosporella*, *Neoacrodontiella*, *Nothoramularia* and *Vanderaaea* formed a well-supported clade (MLB/BPP = 73/1) as a sister group to the well-supported clade (MLB/BPP = 78/1) of the remaining Gomphillaceae that contained lichenized species and two non-lichenized lichenicolous genera (*Corticifraga*, *Taitaia*).

### ﻿Taxonomy

#### ﻿Gomphillaceae Walt. Watson ex Hafellner, Beih. Nova Hedwigia 79: 280. 1984

##### ﻿ Gomphilloideae

Taxon classificationFungiGraphidales

Rivas Plata, Lücking & Lumbsch, Fungal Diversity 52(1): 108. 2012

1607D10D-10EF-5A0E-8565-8C5216B8C2F6

###### Notes.

Nominative subfamily includes current members of the family Gomphillaceae, excluding *Cladosterigma*, *Cytosporella*, *Neoacrodontiella*, *Nothoramularia* and *Vanderaaea*. The development of hyphophores with their diahyphae is a unique feature of this subfamily. Hyphophores with diahyphae are present in many, though not all, members of this lineage (Ferraro 2004; Lücking et al. 2004; Xavier-Leite et al. 2022, 2023).

##### ﻿ Cladosterigmoideae

Taxon classificationFungiGraphidales

Piątek, Stryjak-Bogacka & Czachura subfam. nov.

5697C85D-A201-509C-AB0B-29324B8E92D1

857024

###### Etymology.

Named after the genus *Cladosterigma*.

###### Description.

Non-lichenized, plant associated or fungicolous fungi. Conidiomata sporodochial, synnematal, eustromatic or conidiophores arising directly from hyphae. Conidiophores hyaline, smooth, subcylindrical, conical, ampulliform or subglobose, branched or not, with terminal and/or intercalary conidiogenous cells, sometimes reduced to conidiogenous cells. Conidia solitary or rarely in chains, hyaline, smooth, 0–1-septate, ellipsoid, fusoid or subcylindrical. Sexual morph undetermined [based on generic descriptions in Sutton 1980; Guterres et al. 2020; Crous et al. 2019a, 2021, 2023].

###### Type genus.

*Cladosterigma* Pat.

###### Notes.

This subfamily includes genera *Cladosterigma*, *Cytosporella*, *Neoacrodontiella*, *Nothoramularia* and *Vanderaaea*. Members of this subfamily are different morphologically (absence of hyphophores with diahyphae), phylogenetically (distinct, sister lineage in molecular analyses) and ecologically (non-lichenized and non-lichenicolous species) from representatives of the nominative subfamily.

##### ﻿ Cytosporella fuligomixta

Taxon classificationFungiGraphidales

Piątek, Stryjak-Bogacka & Czachura sp. nov.

97681508-4D2D-5567-A418-9B6D1752CE5E

857025

Figs 2 , 3 , 4

###### Etymology.

Name refers to the isolation of this fungus from sooty mould communities.

**Figure 2.**
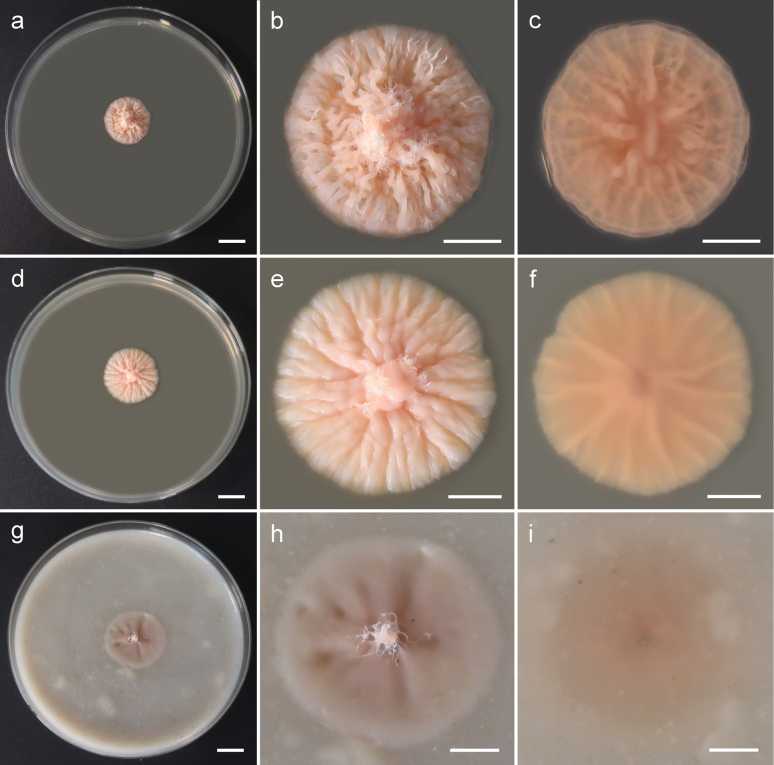
Morphology of cultures of *Cytosporellafuligomixta* (strain G107 = CBS 152343) after 4 weeks of growth at 15 °C: **a–c** general view, upper side and reverse side of colony on MEA **d–f** general view, upper side and reverse side of colony on PDA**g–i** general view, upper side and reverse side of colony on OA. Scale bars: 10 mm (a, d, g); 5 mm (**b, c, e, f, h, i**).

###### DNA barcodes.

ITS (PQ001666), LSU (PQ001665), mtSSU (PP999744), *rpb2* (PP997507) and *tef1* (PP997508).

**Figure 3.**
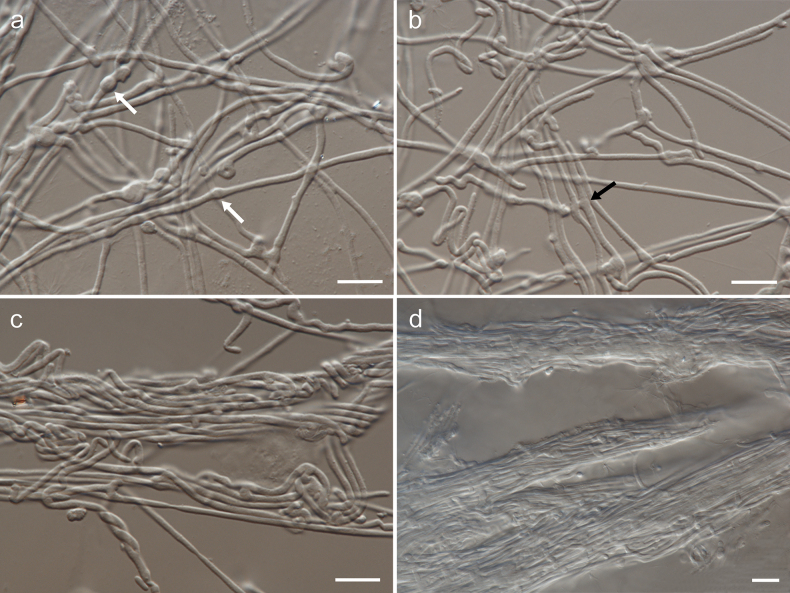
Morphology of *Cytosporellafuligomixta* on MEA (strain G107 = CBS 152343): **a, b** hyphae, white arrows show swellings and black arrow shows anastomosing hyphae **c** intertwined hyphae **d** hyphal fascicles. Scale bars: 10 µm.

###### Typus.

Poland • Małopolska Province, Kraków County: Kraków-Czyżyny (Park Lotników), municipal greenery (city park), isolated from sooty mould community on *Quercusrobur* leaves, 10 Oct. 2018, leg. M. Piątek, W. Bartoszek & P. Czachura (holotype KRAM F-59995; culture ex-type G107 = CBS 152343).

**Figure 4.**
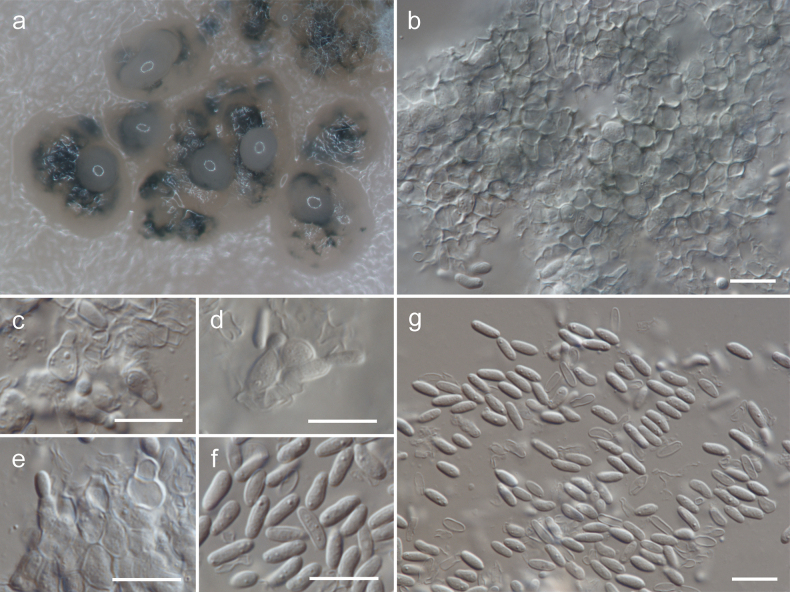
Morphology of *Cytosporellafuligomixta* on OA (strain G107 = CBS 152343): **a** conidiomata **b** wall of textura angularis **c–e** conidiophores **f, g** conidia. Scale bars: 10 µm.

###### Description.

Mycelium composed of sparsely branched, septate, hyaline, straight to curved, thin-walled hyphae, 1.0–1.5 µm wide; sometimes with swellings, 2.0–3.0 µm wide. Hyphae sometimes anastomose, intertwine or form fascicles [description on MEA]. Conidiomata flat, erumpent, separate, eustromatic, brown, disintegrating at the top during maturation, up to 500 µm diam, exuding a creamy conidial mass, partly enclosed by a wall of greenish-olive textura angularis. Conidiophores reduced to conidiogenous cells lining the inner cavity, hyaline, smooth, ampulliform or subcylindrical, phialidic, 3.5–8 × 3–5 µm. Conidia solitary, aseptate, hyaline, smooth, cylindrical, rarely slightly allantoid, apex obtuse, base bluntly rounded, (4–)5–8.5 × 2–2.5(–3) µm [description on OA].

###### Culture characteristics.

Colonies on MEA erumpent, spreading, convex, rosaceous, reaching 4 mm diam after 2 weeks at 6 °C, 9 mm diam after 2 weeks at 15 °C and 6 mm diam after 2 weeks at 25 °C, reaching 6 mm diam after 4 weeks at 6 °C, 18 mm diam after 4 weeks at 15 °C and 8 mm diam after 4 weeks at 25 °C, surface cerebriform, with sparse aerial mycelium, margin finely crenate. Reverse rosaceous. Colonies on PDA erumpent, spreading, umbonate, slimy rosaceous, reaching 4 mm diam after 2 weeks at 6 °C, 11 mm diam after 2 weeks at 15 °C and 4 mm diam after 2 weeks at 25 °C, reaching 7 mm diam after 4 weeks at 6 °C, 20 mm diam after 4 weeks at 15 °C and 7 mm diam after 4 weeks at 25 °C, surface with radial furrows starting from centre towards margin, with sparse aerial mycelium, margin finely crenate. Reverse rosaceous. Colonies on OA spreading, flat, rosaceous, reaching 4 mm diam after 2 weeks at 6 °C, 10 mm diam after 2 weeks at 15 °C and 5 mm diam after 2 weeks at 25 °C, reaching 8 mm diam after 4 weeks at 6 °C, 22 mm diam after 4 weeks at 15 °C and 5 mm diam after 4 weeks at 25 °C, surface with indistinct radial furrows starting from the centre towards the margin, without aerial mycelium, margin entire. Reverse rosaceous.

###### Notes.

*Cytosporellafuligomixta* is well delimited morphologically and ecologically from four other *Cytosporella* species described on *Quercus* hosts. These are *Cytosporellamendax*, *C.pisiformis*, *C.quercus* and *C.sphaerosperma*. All of them were described from branches or wood of *Quercus* sp. or *Quercusrobur* and differ from *C.fuligomixta* in shape and sizes of conidia. The conidia are globose-ellipsoid, hyaline, 4–5 × 3.5–4 µm in *C.mendax* (Saccardo 1884; Saccardo and Roumeguère 1884), globose, yellowish, 3–4 µm in *C.pisiformis* (Saccardo 1884), perfectly globose, hyaline, 9–12 µm in *C.quercus* (Saccardo and Sydow 1902), and globose and hyaline in *C.sphaerosperma* (Saccardo 1884).

Other than being phylogenetically distinct, *Cytosporellafuligomixta* differs also morphologically from three sequenced species of this genus: *C.calamagrostidis*, *C.chamaeropis* and *C.juncicola* (Crous et al. 2019b, 2024). *Cytosporellacalamagrostidis* described from old leaves of *Calamagrostisarenaria* has slightly shorter conidia, (5–)6–7 µm long (Crous et al. 2024), *C.chamaeropis* described from rotten *Chamaeropshumilis* has globose conidia (Passerini 1888) and *C.juncicola* described from culms of *Juncuseffusus* has slightly shorter and narrower conidia, (4–)5–6(–7) × 2 µm (Crous et al. 2019b).

## ﻿Discussion

*Cytosporella* is an understudied genus without modern revision and only with few available DNA sequence data (Sutton 1980; van der Aa et al. 2001; Crous et al. 2019b, 2024; Li et al. 2020). The type species *Cytosporellasycina* has not been sequenced yet, making current taxonomy of the genus tentative. However, all sequenced species of *Cytosporella* form monophyletic lineage. All species assigned to this genus in Index Fungorum (2024) are described from different host plants or differ morphologically if described from the same host plant, which suggests that *Cytosporella* species might be host specific. Currently, sparse DNA sequence data does not exclude host specialization of members of this genus. *Cytosporellafuligomixta* described here from the sooty mould community on *Quercusrobur* leaves is well delimited morphologically and ecologically from four other species described on *Quercus* hosts.

The phylogenetic analyses including sequences of two loci (LSU, mtSSU) showed that sequenced species of *Cytosporella*, together with four other non-lichenized, plant associated or fungicolous genera, namely *Cladosterigma*, *Neoacrodontiella*, *Nothoramularia* and *Vanderaaea*, form sister group to lichenized and lichenicolous fungi in the family Gomphillaceae. Thus, these five genera belong to the family Gomphillaceae and order Graphidales and not to the family Acarosporaceae and order Acarosporales where they were previously included in most studies (Crous et al. 2019a, 2019b, 2021, 2023). The placement of *Cladosterigma* inside the Gomphillaceae was previously reported by Guterres et al. (2020). Recently, Crous et al. (2024) in a phylogenetic tree based on LSU sequences showed placement of these genera (except *Vanderaaea* that was not included in their analyses) inside the Gomphillaceae that was assigned to Ostropales. Most lichenized clades within Ostropales s.l. are also recognised as distinct orders (Graphidales, Gyalectales, Odontotrematales, Ostropales s.str. and Thelenellales) (Kraichak et al. 2018; Lücking 2019). Therefore, if that concept is accepted, *Cladosterigma*, *Cytosporella*, *Neoacrodontiella*, *Nothoramularia* and *Vanderaaea* belong to the family Gomphillaceae and order Graphidales. Moreover, the current analyses showed for the first time that they form distinct, sister lineage to remaining, mostly lichenized genera and species.

Biologically and ecologically, the above mentioned lineage of Gomphillaceae represents a coherent group of non-lichenized species occurring on plants or other fungi. The genus *Cladosterigma* contains only one species *Cladosterigmaclavariellum* that is fungicolous hyphomycete (hyperarasite) occurring on *Phyllachora* species infecting *Eugenia* species (Myrtaceae) in Paraguay and Brazil (Seifert and Bandoni 2001; Guterres et al. 2020). *Cytosporella*, as stated above, includes species forming eustromatic conidiomata and is associated with diverse host plants. The genus *Neoacrodontiella* is typified with *Neoacrodontiellaeucalypti* that produces conidiophores aggregated in sporodochia and occurs on leaves of *Eucalyptusurophylla* (Myrtaceae) in Malaysia (Crous et al. 2019a). The only known species in the genus *Nothoramularia*, namely *Nothoramulariaragnhildianicola*, is a fungicolous hyphomycete (hyperparasite) occurring on cercosporoid *Ragnhildianaferruginea*, which in turn is parasitic on *Artemisiavulgaris* (Asteraceae) in Germany (Crous et al. 2023). *Vanderaaea* is typified with *Vanderaaeaammophilae* that forms sporodochial conidiomata with curved, 0-1-septate conidia and occurs on dead leaves of *Ammophilaarenaria* (Poaceae) in the Netherlands (Crous et al. 2021).

So far, the family Gomphillaceae included mostly foliicolous lichenized fungi, which are predominantly known from the tropics (Xavier-Leite et al. 2022, 2023, 2024). Lichenized fungi from this family are unique in that they form a special type of asexual conidiomata called hyphophores, which produce diahyphae (Ferraro 2004; Lücking et al. 2004; Xavier-Leite et al. 2022, 2023). The family also contained three lichenicolous genera *Corticifraga*, *Paragyalideopsis* and *Taitaia* (Pino-Bodas et al. 2017; Suija et al. 2018; Lücking and Kalb 2002; Xavier-Leite et al. 2022, 2024; Diederich et al. 2024). Current confirmation or inclusion of non-lichenized, plant associated or fungicolous genera *Cladosterigma*, *Cytosporella*, *Neoacrodontiella*, *Nothoramularia* and *Vanderaaea* enlarge the concept of this predominantly lichenized family Gomphillaceae. Due to phylogenetic, morphological (notably: absence of hyphophores with diahyphae) and ecological characteristics a new subfamily Cladosterigmoideae is described for these five non-lichenized genera. It is worthy to note that within Graphidales a very similar situation is found in the lichenized family Graphidaceae, where two non-lichenized genera (*Furcaspora*, *Rubikia*) are now included therein as subfamily Rubikioideae (Cáceres et al. 2020), Additionally, non-lichenized genus *Papilionovela* is a member of the core Graphidaceae (Cáceres et al. 2020). Thus, Gomphillaceae and Graphidaceae, along with still other families of the subclass Ostropomycetidae (e.g. Stictidaceae), constitute good models to study transitions between lichenized and non-lichenized lifestyles.

## Supplementary Material

XML Treatment for Gomphilloideae

XML Treatment for Cladosterigmoideae

XML Treatment for Cytosporella fuligomixta

## ﻿Additional information

### Author ORCIDs

Marcin Piątek https://orcid.org/0000-0002-4968-2861

Monika Stryjak-Bogacka https://orcid.org/0000-0003-2845-9975

Paweł Czachura https://orcid.org/0000-0002-3562-8776

### Data availability

The data that support the findings of this study are available in GenBank (https://www.ncbi.nlm.nih.gov/genbank/) and in culture collections and fungal herbarium, as shown in Table 1 and the text.
